# Acquired Aorto-Right Ventricular Fistula following Transcatheter Aortic Valve Replacement

**DOI:** 10.1155/2015/608539

**Published:** 2015-03-26

**Authors:** Muhammad Tariq Shakoor, Ashequl M. Islam, Samia Ayub

**Affiliations:** ^1^Baystate Medical Center, Tufts University School of Medicine, Springfield, MA 01199, USA; ^2^Division of Cardiology, Baystate Medical Center, Tufts University School of Medicine, Springfield, MA 01199, USA; ^3^Department of Pulmonary and Critical Care, University of Arkansas for Medical Sciences, Little Rock, AR 72205, USA

## Abstract

Transcatheter aortic valve replacement (TAVR) techniques are rapidly evolving, and results of published trials suggest that TAVR is emerging as the standard of care in certain patient subsets and a viable alternative to surgery in others. As TAVR is a relatively new procedure and continues to gain its acceptance, rare procedural complications will continue to appear. Our case is about an 89-year-old male with extensive past medical history who presented with progressive exertional dyspnea and angina secondary to severe aortic stenosis. Patient got TAVR and his postoperative course was complicated by complete heart block, aorto-RV fistula, and ventricular septal defect (VSD) formation as a complication of TAVR. To the best of our knowledge, this is the third reported case of aorto-RV fistula following TAVR as a procedural complication but the first one to show three complications all together in one patient.

## 1. Introduction

Aortic valve replacement is the mainstay treatment for symptomatic aortic stenosis (AS). In properly selected patients, surgery offers substantial improvement in symptoms and life expectancy. However, aortic valve surgery has significant risk in patients with multiple comorbidities, and in a small fraction of patients it can be of “extreme” risk. Catheter-based techniques for aortic valve implantation provide an alternative method for treating AS patients with unacceptably high estimated surgical risks [1]. Transcatheter aortic valve replacement (TAVR) techniques are rapidly evolving, and results of published trials suggest that TAVR is emerging as the standard of care in certain patient subsets and a viable alternative to surgery in others. As TAVR is a relatively new procedure and continues to gain its acceptance, rare procedural complications will continue to appear [2]. Aorto-right ventricular (aorto-RV) fistula has been reported as a complication of aortic valve replacement surgery but to the best of our knowledge there are only a couple of case reports with TAVR [3]. We report a case with complete heart block, aorto-RV fistula, and ventricular septal defect (VSD) formation as a complication of TAVR.

## 2. Case Presentation

**Figure 1:**
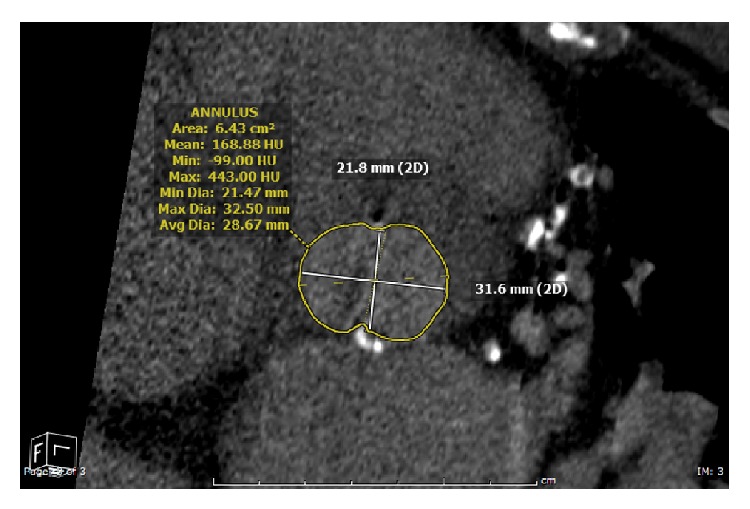
CT determined cross-sectional aortic annular dimensions.

An 89-year-old male with a prior history of laryngeal cancer status after surgical resection and radiation therapy with residual dysphagia, coronary artery disease status after coronary artery bypass grafting in 1992, severe aortic stenosis status after balloon valvuloplasty in 2013, pulmonary fibrosis, COPD on 2-liter continuous home O_2_ therapy, and TIA presented in our clinic with several years of progressive dyspnea and angina with exertion. He was admitted in the hospital at least five times over the past one year with chest pain and dyspnea. Diagnostic cardiac catheterization was done showing three out of four grafts being patent (SVG-RPLV completely occluded). Last year, he underwent balloon aortic valvuloplasty with a 22 mm balloon for severe aortic stenosis and the patient reported partial relief of symptoms for approximately six months after the procedure but now the symptoms are progressively getting worse. Transthoracic echocardiogram (TTE) showed concentric left ventricular hypertrophy, left ventricular ejection fraction of 60%, right ventricle mildly dilated with preserved systolic function, pulmonary artery systolic pressure of 34 mmHg, heavily calcified aortic valve annulus, mean pressure gradient of 68 mmHg, velocity of 517 cm/sec, and aortic valve area of 0.62 cm^2^. Cardiothoracic surgery team evaluated him for aortic valve replacement but the surgeon felt that patient is at very high risk for open-heart surgery. Per our TAVR team, his aortic valve annulus was too big (anteroposterior diameter 2.6 cm and mediolateral diameter of 2.8 cm per transesophageal echocardiogram, Figure 1) for commercially available Core Valve (Medtronic, USA). Ultimately, our TAVR team used a 29 mm Edward Sapien valve via transfemoral access. The procedure was complicated by complete heart block requiring temporary pacemaker backup. A permanent pacemaker was placed after 24 hours. Repeat TTE showed moderately dilated right ventricle with moderately reduced systolic function, pulmonary artery systolic pressure of 40 mmHg, Sapien prosthesis in aortic position with mild perivalvular leak and mean gradient of 9 mmHg, moderate sized continuous (systolic and diastolic phases) shunt which communicates with the aortic root around the prosthesis into the RV (aorto-RV shunt), and pulmonary flow (Qp) to systemic flow (Qs) ratio of 1.39. The patient was managed conservatively with close follow-up. At 30-day follow-up, the patient remained moderately to severely dyspneic. Patient was offered percutaneous closure of the aorto-RV fistula using devices like septal occluder (Amplatzer device) but patient refused it. His repeat TTE showed worsening of aorto-RV fistula and it also showed new small membranous VSD (see Video 1 in the Supplementary Material available online at http://dx.doi.org/10.1155/2015/608539), which was not visible on the first postprocedural TTE. His condition progressively got worse. He changed his code status to comfort measures only and passed away in a couple of days.

## 3. Discussion

**Figure 2:**
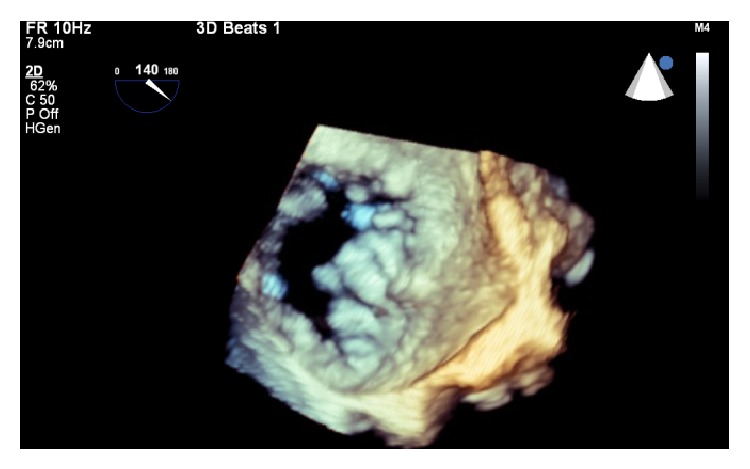
TEE three-dimensional view of aortic valve showing heavy calcification.

**Figure 3:**
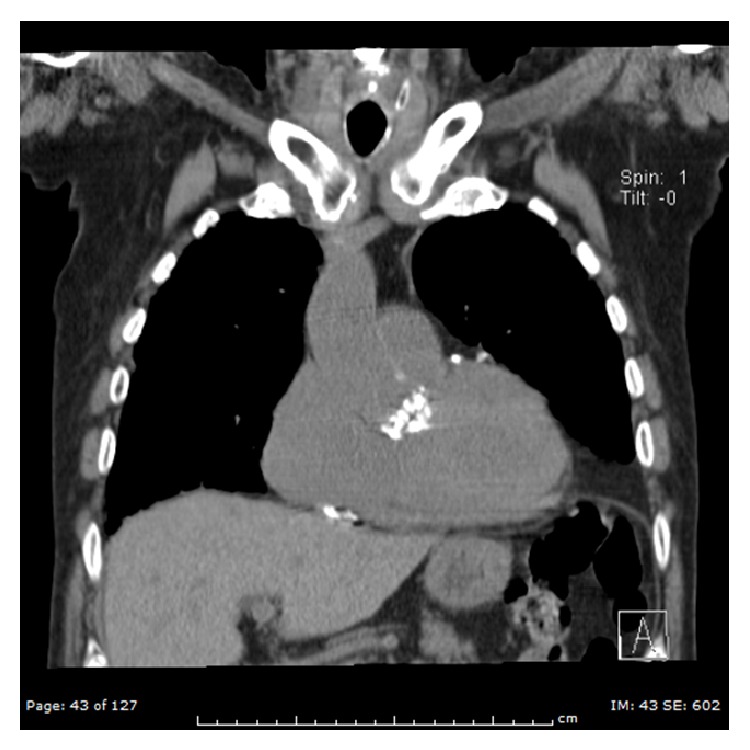
Coronal section of CT chest showing heavily calcified aortic valve.

Complications of TAVR include low cardiac output during and following deployment, annular rupture, vascular complications, myocardial injury, ventricular septal defect, heart block, paravalvular aortic regurgitation, renal failure, and stroke. 2 common procedural complications associated with TAVR have been well studied, whereas knowledge of rare procedural complications and subsequent management rely heavily on experience from case reports. Aortocardiac fistulas are relatively rare. Often, they are a complication of physical/surgical trauma or infective endocarditis [4]. A 2 × 2 cm contact surface between the aorta above the right coronary cusp and the right ventricular outflow tract is the target area for an aorto-right ventricular fistula [4]. In our case the local tissue was heavily calcified and mildly aneurysmal right coronary sinus was seen on aortogram (Figures 2 and 3). So complications may have resulted from displacement of the calcified tissue causing local trauma. The clinical presentation of aorto-RV fistula depends on the size of the shunt. The natural history of aorto-RV fistula after TAVR is unknown but natural history of traumatic aorto-RV fistula was reported by Samuels et al. [4]. In their series of 40 patients, symptoms of heart failure developed at variable intervals and definitive surgical repair was performed in 38 of 40 patients. The mean interval between the time of injury and definitive repair was 1.5 years but in this series 35% of patients had concomitant aortic valve regurgitation; thus it is not clear whether surgery was needed as a result of the aortic valve regurgitation or the large left to right shunt. Nevertheless, spontaneous closure of aorto-RV fistula has not been reported, so careful follow-up of all patients with aorto-RV fistula is prudent. With advances in interventional cardiology, it is now possible to close the aorto-RV fistula percutaneously using devices like septal occluder with three-dimensional TEE guidance in patients who are poor surgical candidates. In our patient 30 days follow up TTE showed a new VSD, which may have resulted from the extension of aorto-RV fistula or perhaps it was missed in first TTE (Video 1).

## 4. Conclusion

To the best of our knowledge, this is the third reported case of aorto-RV fistula following TAVR as a procedural complication. The mechanism responsible for acquired aorto-RV fistula is unknown but it can be related to trauma from displacement of calcified or abnormal tissue or the depth of prosthesis implantation. Conservative management is appropriate in patients without evidence of worsening symptoms and close follow-up is recommended with serial TTE, paying particular attention to right ventricle dimensions and pulmonary artery pressure. If symptoms are getting worse, percutaneous closure of the defect can be considered in this surgically high-risk population.

## Supplementary Material

Video-1: Four chamber view with color-flow Doppler showing a jet from the right aortic sinus to the right ventricular outflow tract, small membranous ventricular septal defect and mild paravalvular aortic regurgitation.
